# The Role of ICL1 and H8 in Class B1 GPCRs; Implications for Receptor Activation

**DOI:** 10.3389/fendo.2021.792912

**Published:** 2022-01-13

**Authors:** Ian Winfield, Kerry Barkan, Sarah Routledge, Nathan J. Robertson, Matthew Harris, Ali Jazayeri, John Simms, Christopher A. Reynolds, David R. Poyner, Graham Ladds

**Affiliations:** ^1^ Department of Pharmacology, University of Cambridge, Cambridge, United Kingdom; ^2^ School of Life and Health Sciences, Aston University, Birmingham, United Kingdom; ^3^ Sosei Heptares, Cambridge, United Kingdom; ^4^ Centre of Sports Health and Life Sciences, Coventry University, Coventry, United Kingdom

**Keywords:** GPCRs, signaling bias, CLR, RAMPs, mutagenesis

## Abstract

The first intracellular loop (ICL1) of G protein-coupled receptors (GPCRs) has received little attention, although there is evidence that, with the 8^th^ helix (H8), it is involved in early conformational changes following receptor activation as well as contacting the G protein β subunit. In class B1 GPCRs, the distal part of ICL1 contains a conserved R^12.48^KLRCxR^2.46b^ motif that extends into the base of the second transmembrane helix; this is weakly conserved as a [R/H]^12.48^KL[R/H] motif in class A GPCRs. In the current study, the role of ICL1 and H8 in signaling through cAMP, _i_Ca^2+^ and ERK1/2 has been examined in two class B1 GPCRs, using mutagenesis and molecular dynamics. Mutations throughout ICL1 can either enhance or disrupt cAMP production by CGRP at the CGRP receptor. Alanine mutagenesis identified subtle differences with regard elevation of _i_Ca^2+^, with the distal end of the loop being particularly sensitive. ERK1/2 activation displayed little sensitivity to ICL1 mutation. A broadly similar pattern was observed with the glucagon receptor, although there were differences in significance of individual residues. Extending the study revealed that at the CRF1 receptor, an insertion in ICL1 switched signaling bias between _i_Ca^2+^ and cAMP. Molecular dynamics suggested that changes in ICL1 altered the conformation of ICL2 and the H8/TM7 junction (ICL4). For H8, alanine mutagenesis showed the importance of E390^8.49b^ for all three signal transduction pathways, for the CGRP receptor, but mutations of other residues largely just altered ERK1/2 activation. Thus, ICL1 may modulate GPCR bias *via* interactions with ICL2, ICL4 and the Gβ subunit.

## Introduction

GPCRs form the single largest protein family in the human genome, and they are also the largest single target for therapeutic agents (1). They all share a common architecture based around seven transmembrane helices (TMs), connected by three intracellular and three extracellular loops (ICLs and ECLs), often with an 8^th^ helix (H8), immediately after TM7, lying parallel to the membrane. They are divided into several families. The largest and best understood is the class A or rhodopsin-like family. The class B1, or secretin-like family is smaller but is made of receptors for physiologically important peptides such as glucagon, corticotrophin-releasing factor (CRF) and members of the calcitonin gene-related peptide (CGRP) family. There are cryo-electron microscope structures for most of these receptors, showing their structure with Gs or other G proteins (2) and crystal or cryo-EM structures are available for the transmembrane domains of the glucagon, glucagon-like peptide-1 (GLP-1), CRF type-1 (CRFR1), CGRP and parathyroid type-1 receptors (3–10).

The means by which class A GPCRs recognise G proteins is now understood in detail for a number of receptors (11). There is also growing understanding of GPCR binding to β-arrestins (12). It has been possible to trace how agonist binding leads to TMs 3 and 6 (and hence ICLs 2 and 3) moving apart, to allow an interaction with the C-terminus of the G protein. It remains unclear how different receptor-ligand combinations select between individual G proteins or β-arrestins, although this is key to understanding biased agonism - the phenomenon whereby agonists produce unique signals at receptors (10). The activation of class B1 GPCRs appears to be broadly similar although they have their own unique motifs; thus, residues at the base of TM2 and its junction with ICL1 function in combination with TM 3 to create a G protein-binding pocket (7–10). The individual microswitches used by class A and class B1 are distinct (13). For class B1 GPCRs, it seems that the key to receptor activation is the full engagement of the agonist peptide N-terminus with the TM domain of the GPCR, which initiates changes at the intracellular face of the receptor (14, 15). ICL1 has been little studied in either GPCR class. Mutations can disrupt cell surface expression (16, 17), indicating the loop is important for structural integrity of the receptor. It is adjacent to ICL2 and in the class A and B1 GPCR structures there is an interaction between it and the proximal region of H8. The junction between H8 and TM7 is sometimes known as ICL4. ICL1 has a role in receptor activation, as revealed by mutations in a range of GPCRs from class A and B1 (18–26). Hydrogen-deuterium exchange indicates that different agonists cause distinctive conformational changes in ICL1, similar to those seen at ICL4 (27). Changes in the ICL1-H8 unit precede movement of TMs 5 and 6 in the μ-opioid receptor, showing that they are an early event in GPCR activation (28, 29); this may be part of a conserved activation mechanism (30). There is also good evidence for ICL1 playing a role in G protein selectivity through splice variants in class B1 GPCRs (31). The mechanism behind the actions of ICL1 is unclear.

Here we study the role of ICL1-H8 unit in the activation of class B1 GPCRs and in particular, the significance of a motif previously referred to as the [K/R]KLH motif (32) within ICL1 which mediates its interactions with H8. We have primarily concentrated on the calcitonin receptor-like receptor (CLR), a receptor we have studied (33, 34), in association with receptor activity-modifying protein 1 (RAMP1), this forms a receptor for CGRP which can also recognize the related peptide adrenomedullin (AM) (35). We have also examined the glucagon receptor (GCGR) and two splice variants of the CRFR1.

## Methods

### Materials

Human CGRP and CRF were purchased from Bachem and human glucagon was purchased from Alta Biosciences. All peptides were made to 1 mM stocks in water containing 0.1% bovine serum albumin (BSA).

### Constructs and Site-Directed Mutagenesis of CLR and GCGR

The CRFR1a and CRFR1b constructs were gifts from Dr. Simon Dowell (GSK, Stevenage, UK). ICL1 mutants of CLR were generated in a pIRES-RAMP1-SNAP-CLR construct in-house by Sosei Heptares and confirmed through Sanger sequencing. ICL1 mutants of the GCGR were generated as for CLR except using a base vector of pcDNA3.1-GCGR-GFP. H8 mutants for CLR and GCGR were generated by using the QuikChange Lightening Site-Directed Mutagenesis Kit (Agilent Technologies) in accordance with the manufacturer’s instructions and sequenced in house at Cambridge University. All constructs were of human receptors and RAMP.

### Transfection and Cell Culture

HEK 293T cells (a gift from Professor Colin Taylor) were cultured in DMEM/F12 GlutaMAX supplemented with 10% fetal bovine serum (FBS), and incubated at 37% in humidified 95% air, 5% CO_2_. Plasmids were transfected into HEK 293T cells using FuGENE HD according to the manufacturer’s instructions using a 1:3 w:v ratio of DNA : FuGENE and cultured for 48 hours prior to assaying.

### Quantification of Mutant GPCR Expression

Cell surface expression of WT and mutant GPCRs was determined in HEK 293 cells *via* flow cytometry as previously described (36). Briefly, after 48 hours, cells were washed three times in fluorescence activated cell sorting (FACS) buffer (PBS supplemented with 1% bovine serum albumin (BSA) and 0.03% sodium azide) before and after 1 hour incubation at room temperature in the dark with appropriate primary antibody; rabbit anti-GCGR (AGR-024, diluted 1:50 (Alomone Labs)) or rabbit anti-SNAP (CAB4255, diluted 1:100, Invitrogen). Cells were then incubated for a further hour at room temperature in the dark in secondary antibody (goat APC-conjugated anti-rabbit IgG polyclonal antibody, diluted 1:150 (ThermoFisher)). Samples were analysed using a BD Accuri C6 flow cytometer (BD Biosciences) Ex. λ 633 nm and Em. λ 660 nm. Mean APC intensity indicated the plasma membrane expression of each GPCR. Data were normalized to the mean APC intensity of cells transfected with WT GPCR as 100% and pcDNA3.1(-) as 0%.

### Measurement of Intracellular cAMP Accumulation

HEK 293 cells expressing WT or mutant GPCR were assayed for cAMP accumulation as previously described (33, 37). cAMP accumulation was measured after 30 minutes stimulation using LANCE^®^ cAMP Detection Kit (Perkin Elmer Life Sciences) on a Mithras LB 940 multimode microplate reader (Berthold Technologies). Data were normalized to the maximal level of cAMP accumulation from cells in response to 100 μM Forskolin (Sigma) stimulation.

### Measurement of Intracellular Calcium Mobilization

Intracellular calcium mobilisation was measured in transfected HEK 293 cells as previously described using Fluo-4/AM and a Mithras LB 940 multimode microplate reader (33). Data were normalized to the maximal intracellular calcium release in response to 10 μM ionomycin.

### Measurement of Phospho-ERK_1/2_ (Thr202/Tyr204)

HEK 293 cells expressing WT or mutant GPCR were serum starved overnight prior to assaying. Cells were then washed and resuspended in Ca^2+^ free HBSS and seeded at a density of 35000 cells per well in 384-well white Optiplates (Perkin Elmer). Cells were stimulated with ligand for 5 min, before lysis using the supplied lysis buffer and assayed for ERK1/2 phosphorylation using the phospho-ERK (Thr202/Tyr204) Cellular Assay Kit (Cisbio). Plates were read using a Mithras LB 940 multimode microplate reader and data normalized to the maximal response to 100 μM phorbol 12-myristate 13-acetate (PMA, Sigma) (34).

### Molecular Dynamics

PDB structures for the inactive and active conformations of CLR complexed with RAMP1 (PDB codes 7KNT and 6E3Y, respectively) and the GCGR (PDB codes 5EE7 and 6WHC, respectively) were obtained from the PDB. Missing atoms were identified from the PDB header and are available in the original PDB file. They were replaced using MODELLER (38) prior to refinement and scoring using Rosetta (39). To reduce errors introduced by loop modelling, ICL2 was excluded from the MODELLER step. Furthermore, no attempt was made to refine existing regions of secondary structure as are as per the original crystallographic file. The best scoring inactive and active conformations of each receptor type were used for an essential dynamics simulation (40). Each protein was embedded in an equilibrated solvated membrane consisting of 280 POPC lipids. NaCl was added at a concentration of 150 mM, with extra Cl^-^ ions added to the solvent to neutralize the system. Protonation states of charged residues were determined using ProPka (41) prior to the simulation start. Initial equilibration simulations (100 ns) were performed using Gromacs (42) at 310 K for both the active and inactive receptor states for each receptor family. The equilibrated structures were then used for an essential dynamics simulation. During the essential dynamics simulations, each inactive conformation had a fixed potential applied to the first eight non rotational/translations eigenvectors that increased in fixed increments per step to drive the system from the inactive to the active state. Simulations were performed with fixed increments of 1.2 × 10^−6^ nm per each simulation step (2 fs). Each simulation was performed 10 times. The amber-ILDN forcefield was used in all simulations. The simulations were combined into an inactive to active trajectory using the best scoring snapshot, using the Rosetta scoring function at each timestep. The snapshots were not chosen, but are equally spaced conformations that describe the trajectory from inactive to active state. The RMSD between snapshots was approximately 0.01 Angstroms.

### Generation of Weblogo’s

GPCR sequences were obtained for 289 human GPCRs in Class A and Class B1 from GPCRdb (https://gpcrdb.org) (43). Sequences were aligned for their ICL1 and H8 using GPCRDB outputs. Weblogo’s were then created using free software from] https://weblogo.berkeley.edu using the frequency method where height of each letter represents the frequency of each amino acid.

### Data Analysis

All pharmacological data was analyzed in GraphPad Prism v9.0 (GraphPad Software, San Diego). Data were fitted to obtain concentration–response curves using either the three-parameter logistic equation to obtain values of E_max_ and pEC_50_ or the operational model of agonism (44) as described previously (33, 34), to obtain efficacy (*τ*) and equilibrium disassociation constant (K_A_) values. Statistical differences were analysed using one-way ANOVA followed by Dunnett’s *post-hoc* (for comparisons amongst more than two groups). Data was normalized to either activity of Wild Type receptor or relative to a system parameter control; 100 μM forskolin stimulation for cAMP accumulation, 10 μM ionomycin for _i_Ca^2+^ mobilizations assays or 10 μM phorbol 12-myristate 13-acetate (PMA) for ERK1/2 activation. The means of individual experiments were combined to generate the concentration-response curves displayed in the figures. Having obtain values for *τ* and K_A_ these were then used to quantify signaling bias as the change in Log(*τ*/K_A_) relative to WT and a reference signaling pathway (ERK1/2) as described previously (34).

## Results

### Sequence Conservation Within ICL1 and H8

**Figure 1:**
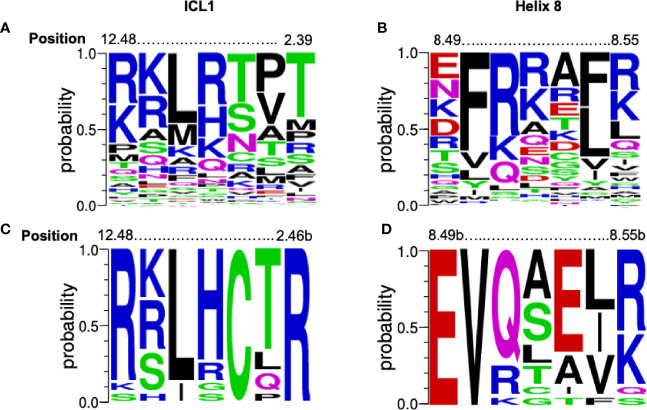
Conservation of the [K/R]KL[R/H]xx[T/R] and EFxxxL motifs. Alignment of the amino acids from 298 human GPCRs (both Class A and B1) for **(A)** ICL1 and **(B)** Helix 8 depicted as a weblogo’s. Position marked using the Ballesteros-Weinstein numbering systems combined. Alignment of the amino acids from human Class B1 GPCR alone for **(C)** ICL1 and **(D)** Helix 8 depicted as a weblogo’s (45). Position marked using the Wootten Class B1 numbering, (46). The height of each letter represents the frequency of each amino acid. Amino acids are colour coded to their chemical properties: polar amino acids (G,S,T,Y,C) are green, neutral (Q,N) are magenta, basic (R,K,H) are blue, acidic (D,E) are red, and hydrophobic (A,V,L,I,P,W,F,M) are black. Figures generated using https://weblogo.berkeley.edu..

There is a high level of conservation in both ICL1 and H8 in all GPCRs (**Figures 1A, B**) and especially Class B1 GPCRs (**Figures 1C, D**). In Class B1 GPCRs only, ICL1 contains an R^12.48^KLR**C**x**R** ^2.46b^ motif that extends into TM2 (using the Wootten Class B1 numbering, 46); the C and R in bold are absolutely conserved. In H8 there is an N^7.61b^G**E** ^8.49b^-**V** *X*xx*X*(R/K)xW motif where *X* is hydrophilic, x is any amino acid and the E and V in bold also absolutely conserved (**Figure 1** and **Figure S1**). The sequences of ICL1 and H8 motifs are more precise than our initial reports (32); here we only consider human class B1 GPCRs, rather than the full class B sequences (adhesion + secretin families).

### Mutational Analysis of the ICL1-H8 Regions of CLR

To understand the function of ICL1 and H8, we first performed an alanine scan centred on the R^12.48^KLRCxR and E^8.49^VXxxX motifs in CLR. The ICL1-H8 motifs (underlined) are represented in the sequences Y^165^FK^167/12.48b^SLSCQR ^173/2.46b^ and N^388/7.61b^GE^390/8.49b^VQAIL ^395/8.54b^. Almost all mutants were expressed at the cell surface at a third or greater than that of WT (**Tables S1, S2** – except I394A), a level which we have previously established that causes little change in agonist potency (47). It should however be noted that we had not checked for effects on efficacy/E_max._ L169A expression was reduced by 72% and there were also sizeable decreases in expression for E390A, I394A, I397Aand L398A and R399A; the expression of L395A and K167A were reduced by 50%.

**Figure 2:**
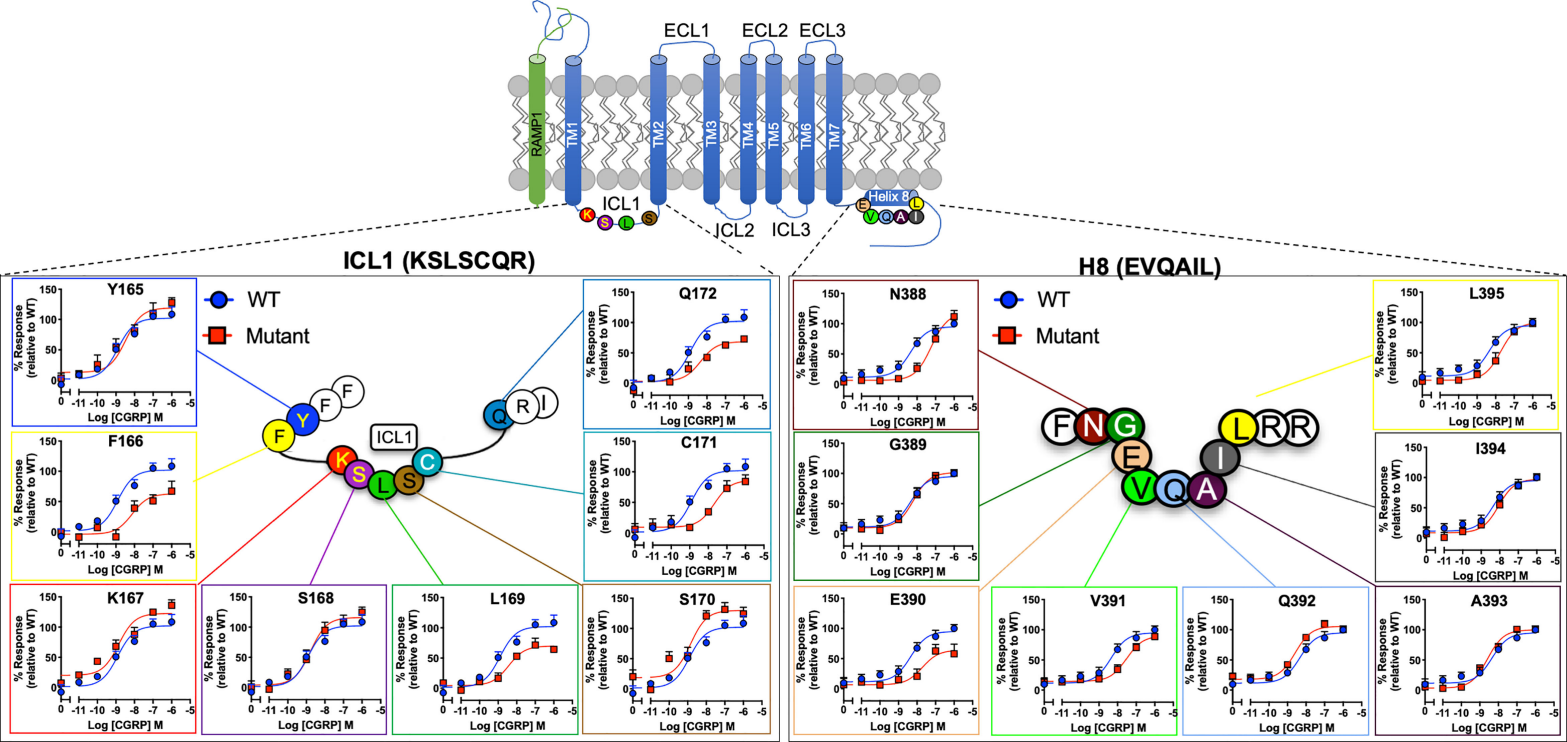
Mutational analysis of residues in ICL1/H8 at the CGRP receptor. Residues in the ICL1 (left) and H8 (right) were mutated to Ala (except A393 where Leu was used) and the effects on cAMP production compared to wild type receptor. Mutant curves are show in red, wild type in blue. All data are mean ± SEM of n repeats where n=minimum of 3 triplicates.

When cAMP production was measured, mutation of F166, L169, C171 and Q172 (**Table S3A** and **Figure 2**) all reduced CGRP potency or E_max_ while S170A slightly increased it. For H8 and the base of TM7 there were significant reductions in pEC_50_ for N388A, E390A (also decreasing E_max_), V391A and W399A (**Table S3A**).

**Figure 3:**
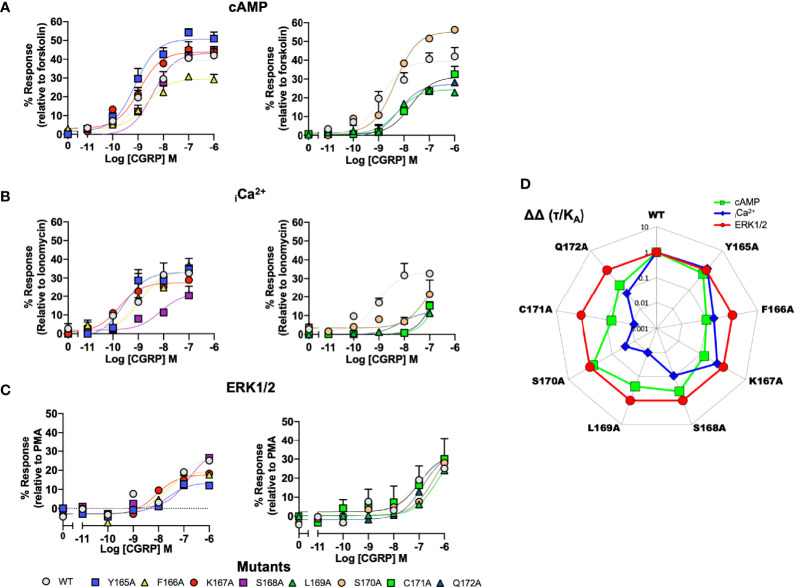
Signaling bias of alanine mutants of ICL1 at the CGRP receptor. Residues in the ICL1 were mutated to Ala (except A393 where Leu was used) and the effects on **(A)** cAMP production, **(B)** elevation of intracellular Ca^2+^, **(C)** pERK were compared to wild type receptor, **(D)** Signaling bias plot was calculated as 10^ΔΔ(*τ*/KA)^ for each mutant and each signaling pathway relative to WT and ERK phosphorylation. Note I394A has not been included in the analysis due to a lack of signaling in the _i_Ca^2+^ and ERK1/2 assays. All data are mean ± SEM of n repeats where n=minimum of 3 triplicates.

To provide a comparison of how the ICL1 mutants might influence downstream signaling activity, we renormalised the cAMP accumulations data shown in **Figure 2** to now account for the maximal level of cAMP accumulation achievable for HEK 293T cells (stimulations with the non-selective adenylyl cyclase activator forskolin), (**Figure 3A** and **Table S3B**). Beyond cAMP accumulation, CLR can also couple to Gα_q/11_ to increase intracellular calcium (33, 48). When this response is measured, the ICL1 mutants show a somewhat different pattern of activity; whilst L169A and C171A again reduce potency, with S168A there was a decrease in apparent affinity calculated from the operational model (**Figure 3B** and **Table S4**). No mutants enhanced coupling. Alanine mutation of the ICL1 appeared to show little overall effect upon CGRP-induced ERK1/2 activation [which was largely independent of Protein Kinase A activation, (34)], with only K167A showing any significant changes; an increase in potency but decrease in E_max_ (**Figure 3C** and **Table S5**).

While the changes in potency for cAMP and _i_Ca^2+^ mobilisation appear to suggest changes in signaling bias profiles, to formally confirm this, and to remove potential confounding issues of system bias, we refitted the data in **Figure 3** using the operational model of receptor agonism (44). Since the ERK1/2 activation seemed to tolerate mutation at any positions in ICL1, all data was normalised to this parameter (**Figure 3D** and **Tables S3B, S4, S5**). This analysis reconfirmed that mutations of residues L169 to Q172 all resulted in a signaling response that was biased away from _i_Ca^2+^ mobilisation and towards cAMP. Moreover, mutations of C171 and Q172 also displayed bias away from cAMP accumulation to a lesser extent than for mobilisation of _i_Ca^2+^.

**Figure 4:**
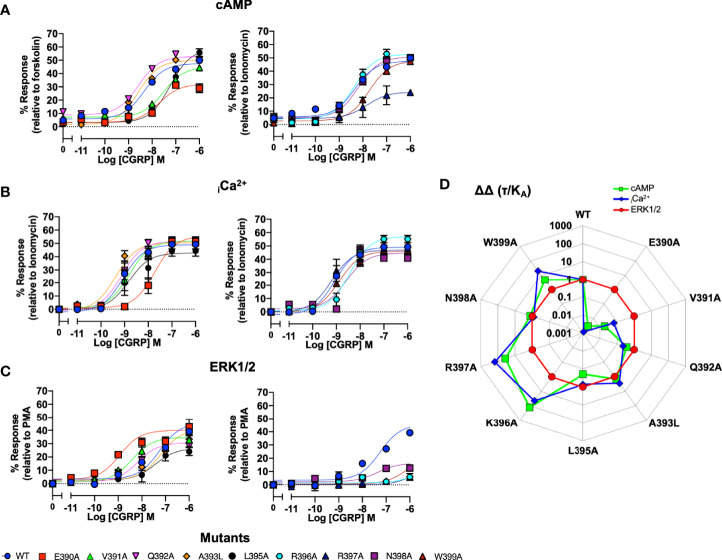
Signaling bias of alanine mutants of H8 at the CGRP receptor. Residues in the H8 were mutated to Ala and the effects on **(A)** cAMP production, **(B)** elevation of _i_Ca^2+^, **(C)** ERK were compared to wild type receptor, **(D)** Signaling bias plot was calculated as 10^ΔΔ(*τ*/KA)^ for each mutant and each signaling pathway relative to WT and ERK1/2 phosphorylation. All data are mean ± SEM of n repeats where n=minimum of 3 triplicates.

We next performed the same array of assays as described for the ICL1 mutants on the CLR H8 mutants. Normalisation of the cAMP accumulation data showed that N388A, E390A, V391A, L395A and W399A all displayed reduced potency (**Figure 4A** and **Table S3B**). For _i_Ca^2+^ mobilisation, only E390A showed a dramatic reduction in potency (**Figure 4B** and **Table S4**). Unlike the ICL1 mutants, mutations at every position within the H8 motif changed either potency (with increases for E390A, V391A, and Q392A) or E_max_ when ERK1/2 activation was quantified (**Figure 4C** and **Table S5**). Analysis using operational parameters confirmed these observations (**Figure 4D** and **Tables S3B, S4, S5**). I394A displayed reduced cell surface expression and failed to generate responses for _i_Ca^2+^ mobilisation or ERK1/2 activation so was excluded from any analysis although it did show a WT cAMP response, for reasons that are not obvious. Thus, the effects of mutation on ICL1 and H8 differ depending on which coupling pathway is examined and ERK1/2 activation is much more sensitive to mutations in H8 than cAMP or _i_Ca^2+^ elevation. These differences are highlighted in the bias plots for ICL1 and H8.

**Figure 5:**
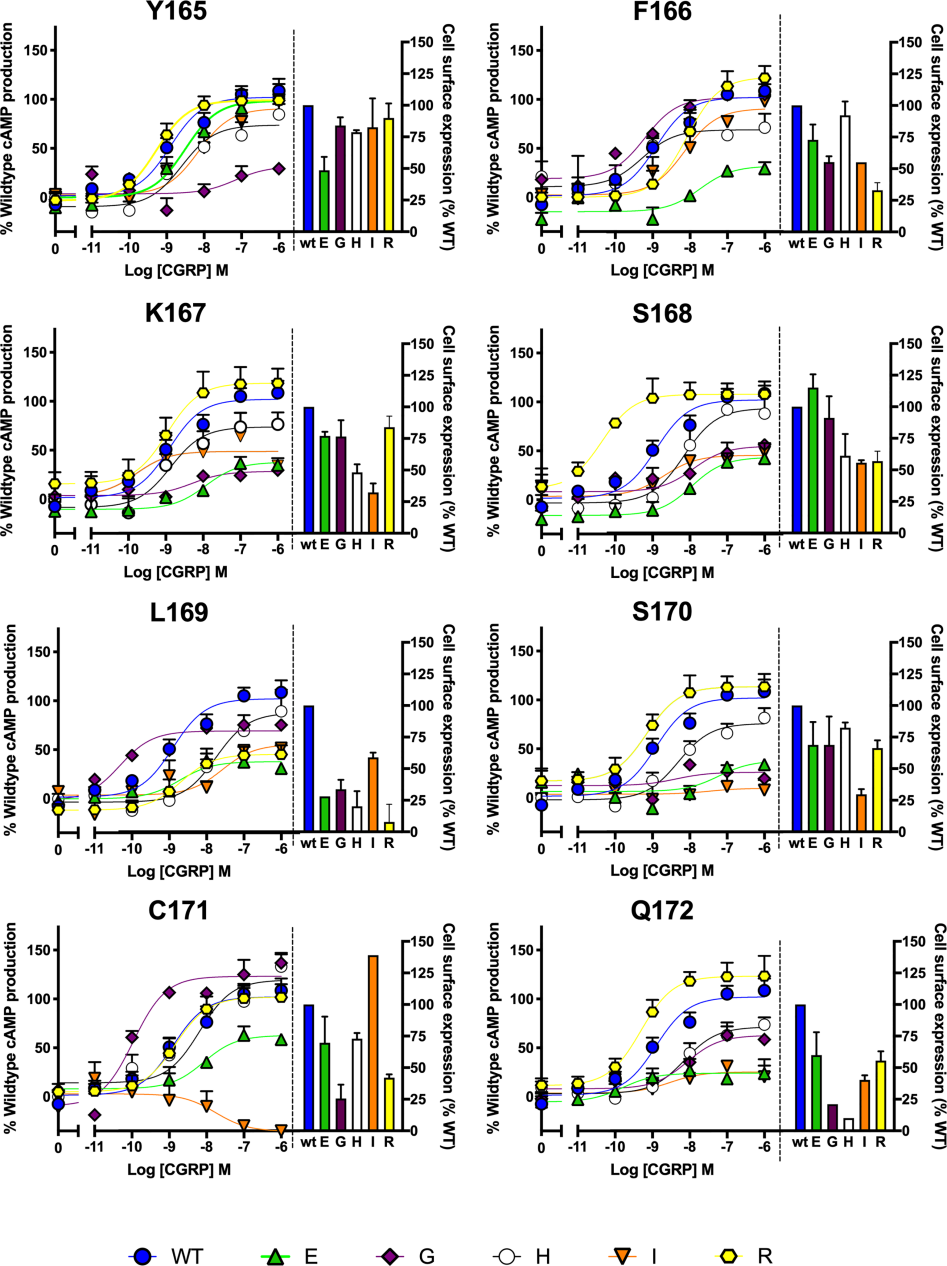
Mutagenic analysis of residues in ICL1 at the CGRP receptor. Residues in the ICL1 were mutated to a wide range of non-complementary amino acids (as indicated) and the effects on cAMP production compared to wild type receptor (concentration-response curves). Cell surface expression for each mutant was quantified and normalized to the wild type receptor. All data are mean ± SEM of n repeats where n=minimum of 3 triplicates.

After preliminary experiments using saturation mutagenesis of ICL1 on cAMP production, the effect of glutamate, arginine, histidine, glycine and isoleucine were explored in detail, to examine the effect of charge and size at each position. This showed that substitutions at every position can alter receptor activation and/or receptor expression (**Figure 5**). Several interesting features appear from this extended data. Substitution by R within the motif is well tolerated apart from at L169; S168R increases CGRP potency by almost 100-fold and there was a small increase in Emax with Q172R. Substitution by H was best tolerated at C171 and replacement by E gives particularly large decreases in activity at every position apart from F165. Replacement of L169 by any residue apart from I reduces expression by at least ^2^/_3_ compared to WT. Glycine substitution, generally deleterious, increases potency at L169 (albeit with a reduced E_max_) and C171.

### Extension of Alanine Scan of ICL1 to the GCGR

**Figure 6:**
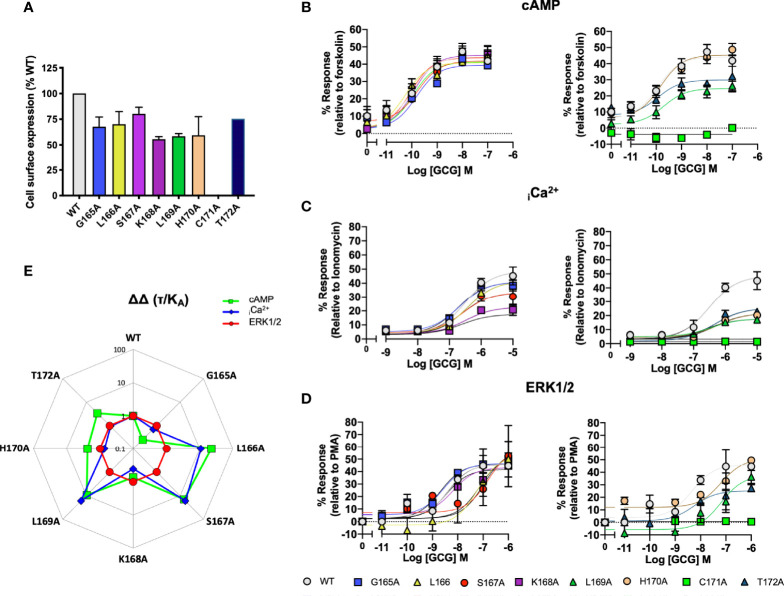
Signaling bias of alanine mutants of ICL1 at the Glucagon receptor. Residues in the ICL1 were mutated to Ala and the effects on **(A)** Cell surface expression, normalized to wild type GCGR, **(B)** cAMP production, **(C)** elevation of _i_Ca^2+^, **(D)** ERK1/2 were compared to wild type receptor, **(E)** Signaling bias plot was calculated as 10^ΔΔ(*τ*/KA)^ for each mutant and each signaling pathway relative to WT and ERK1/2 phosphorylation. All data are mean ± SEM of n repeats where n=minimum of 3 triplicates.

In the GCGR, the sequence of ICL1 is G^165/1.61b^L^12.47b^ SKLHCTR and the corresponding H8 sequence is NE^406/8.49b^VQSEL (underlined residues are the motifs, as shown for CLR in section 3.1 and 3.2). This is a more typical H8 sequence as 8.53b is E in 12/15 human class B GPCRs. To probe the differences/conservation of regions of activity, we focussed upon mutations of the ICL1 region of the GCGR. C171A failed to express and so was without activity in all signaling assays; K168A, L169A and H170A showed a 50% reduction in expression. For the other mutants, cell-surface expression was not changed (**Figure 6A**). Alanine mutation of L169^12.50^ and T172^12.52^ reduced the extent of cAMP production; the other mutants that expressed were without effect on this pathway (**Figure 6B**). By contrast, both elevation of _i_Ca^2++^ mobilisation and (in contrast to the CLR) ERK1/2 signaling were far more sensitive to alanine substitution throughout ICL1 (**Figures 6B–E** and **Table S6**).

Immediately outside ICL1, R173^2.46b^A reduced the potency of glucagon, almost 100-fold, on cAMP accumulation (pEC_50_ WT, 9.09 ± 0.1; pEC_50_ R173A, 7.14 ± 0.1, p>0.01, n= 5). A limited characterisation of H8 was carried out, focussing on the two charged residues E406^8.49b^ and E410^8.543b^. The only effect on cAMP production was a very small increase in basal activity seen with E406A (WT 7.6 ± 1.1% of response to forskolin, E406A, 11.1 ± 1.5%, p>0.05, n=5). With the double mutant E406AE410A, the basal was further increased to 19.6 ± 1.4% (p>0.01), again with no change in potency. However, for E406A, expression was 53.8 ± 5.8% of WT and this was further reduced to 21.5 ± 1.3% with E406AE410A, suggesting an increase in the effectiveness of coupling to Gs given the increase in basal and the maintenance of potency.

### ICL1 in the CRF-Type 1 Receptor (CRFR1)

**Figure 7:**
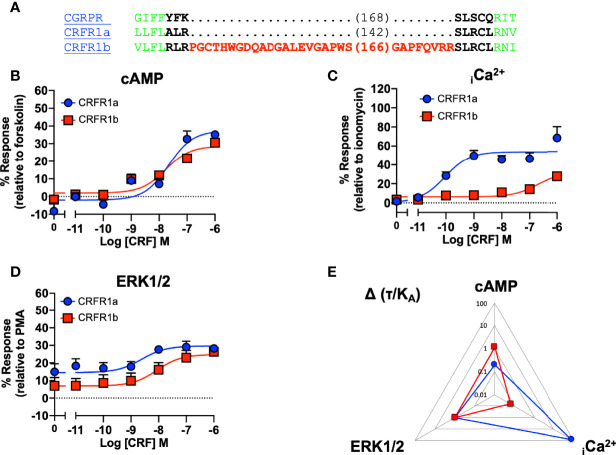
Signaling bias of splice variants of ICL1 at the CRF receptor. **(A)** Sequence of CRFR1 splice variants, with CGRPR as reference. Splice variants of the CRFR1 were compared for **(B)** cAMP production, **(C)** elevation of _i_Ca^2+^, **(D)** ERK1/2 phosphorylation. **(E)** Signaling bias plot was calculated as 10^Δ(*τ*/KA)^ for each mutant and each signaling pathway relative to ERK1/2 phosphorylation. All data are mean ± SEM of n repeats where n=minimum of 3 triplicates.

Our data suggested that ICL1 might have a role in determining G protein preference of Class B1 GPCRs. The CRFR1 exists in two splice forms (1a and 1b) which differ due to the presence of a 29 amino acid insert in the RKLR motif which does not influence cell surface expression (49). Analysis of the signaling profiles suggests that the CRF1a receptor, which lacks the insert, is biased towards Ca^2+^ signaling, which is consistent with the motif being important for G protein specificity (**Figure 7** and **Table S7**).

### Molecular Dynamics Simulation of ICL1 During Activation of the CGRP Receptor

**Figure 8:**
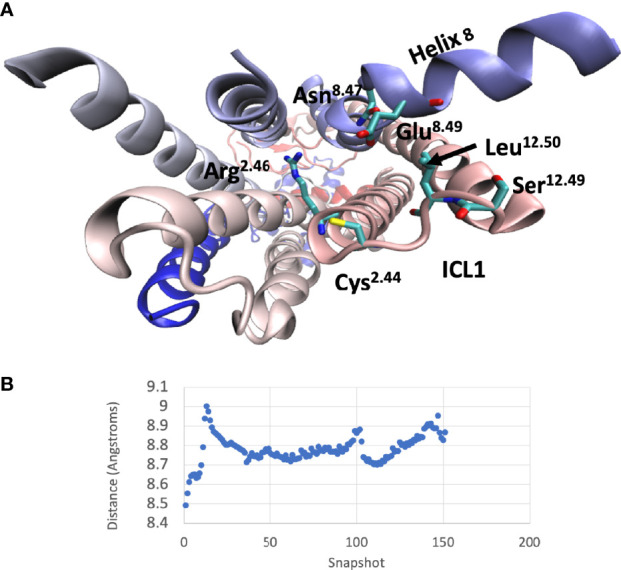
Interactions of ICL1 during a molecular dynamics simulation of CLR. **(A)** Cytoplasmic view of CLR showing the main sidechains involved in interactions with ICL1. **(B)** Variation of the minimum distances between the centres of mass of L169-E390 during the molecular dynamics simulation.

A comparison between the inactive and active CGRP and GluR structures (the latter in complex with both Gs and Gi) indicated that ICL1 only underwent subtle changes during the process of receptor activation. To further understand why mutations throughout this region can change activation, a molecular dynamic simulation was used to study the inactive-active transition in the CGRP receptor (**Figure 8** and **Videos S1A, B**). Early during the simulation, the proximal region of ICL1 underwent a flexing movement centred on S168^12.49^. This subtly changes the orientation of ICL1 with respect to both ICL2 and H8. This was followed by movements of ICL2. Around the middle of the simulation, N388^7.61b^ and E390^8.49^ in ICL4 move towards L169^12.50^. There is then a bending of the distal part of TM1 but ICL1 with H8 and ICL2 move largely as a rigid body as a consequence of this. At the very end of the simulation there are further movements of N388 and E390, coupled eventually to a change in the side-chain orientation of R173. At this stage, the G protein binding pocket is now fully open. The distance between L169 in ICL1 and E390 in H8 reflects these changes (**Figure 8B**). Broadly similar movements were seen during a molecular dynamics simulation of the glucagon receptor, with both early and late movements in ICL1 (**Video S2**).

## Discussion

The role of ICL1 in mediating signal transduction is poorly explored. In this paper, we confirm evidence (32) evidence for a structural motif in the loop which is an important player in determining the specificity of G protein-coupling. It is possible to interpret some of the data using the current cryo-EM and crystal structures with further insight coming from molecular dynamics simulations. We have also used mutagenesis to explore the role of individual residues. Mutagenesis data needs to be interpreted with care as the mutants themselves can change the structure of a protein; however, it can be useful to explore interactions predicted from structural and modelling studies.

**Figure 9:**
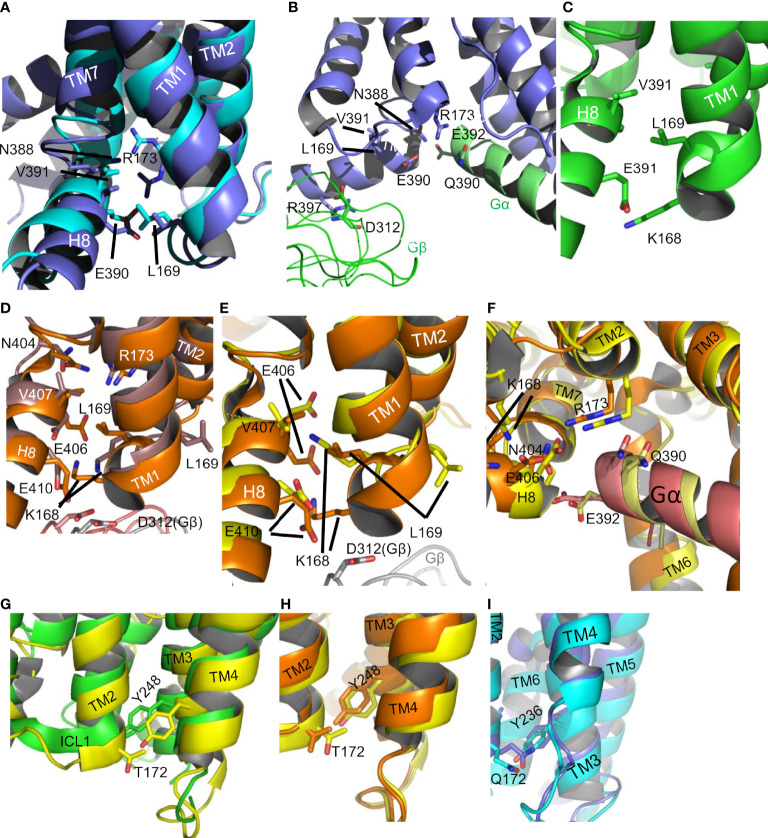
The ICL1-H8 interface in CGRP. **(A)** The interface (6E3Y, active, slate; 7KNT inactive, cyan). **(B)** Contacts of the active CGRP (6E3Y, active, slate) receptor with Gαs (cartoon helix, green) and Gβ (ribbon, green). **(C)** The ICL1-H8 interface in GCGR (5EE7 inactive, green). **(D)** The ICL1-H8 interface in GCGR ((6WPW active Gαs, orange/6LML active Gαi, brown)). In the inactive crystal structures 4L6R, 5YQZ, 5XEZ and the G protein bound cryo-em structures 6WHC, 6LMK, and 6LML, there is a rotation in the middle of the ICL1 so that L^12.50^ points upwards between TM1 and TM2 and K^12.49^ points to H8. E^8.49^ and E^8.53^ can interact with the side chain of K^12.49^, perhaps helping to keep H8 engaged with ICL1. In the inverse-agonist stabilised crystal structure 5EEZ and the Gαs-bound cryo-em 6WPW, L^12.50^ points to H8 and K^12.49^ is beneath the receptor. D312 of Gβ is close to IL1 in both structures. **(E)** The ICL1-H8 interface in GCGR (6WPW active Gs, orange/6WHC active Gαs, yellow). The variation of the K168 orientation is indicative of water mediated interactions. **(F)** Gαs contacts in GCGR for 6WPW active, orange and 6WHC active, yellow. **(G)** The ICL1-ICL2 interface in the GCGR (5EE7 inactive). **(H)** The ICL1-ICL2 interface in the GCGR (6WPW active Gαs, green/6WHC active Gi, orange). **(I)** The ICL1-ICL2 interface in the CGRP (6E3Y, active, cyan).

At first sight, the claim that ICL1 is important in modulating G protein signaling may seem surprising. The loop is usually short and could be considered simply as a linker between TMs 1 and 2. It makes no direct contact with the a subunits of G proteins. Furthermore, its conformation in G protein associated GPCRs is very similar to that of the apo-receptors. However, it has several interfaces in Class B1 GPCRs, as seen from structures (3–5, 8, 9, 50–55) (**Figure 9**). The distal end of the loop faces towards TM3/4 and ICL2. It can indirectly influence ICL3 *via* changing the position of H8. In the inactive receptor, it can contact a string of bound water molecules that fit between TMs 2 and 7, at least in the high resolution GCGR structure, 5EE7 (2.5Å). The H8-TM7 junction (ICL4) makes important contacts with the G protein when it binds to the receptor. ICL1 contacts the Gβ subunit of the bound G protein (**Figure 9E**) and R^2.46b^ faces the Gα subunit (**Figures 9B, F**). The contacts between ICL2 and, especially, ICL4 are of considerable potential significance as it is likely that these are key determinants of G protein selectivity (2, 56). ICL4 (between TM7 and H4) directly contacts the H5 helix of Gα subunits, which enters the cavity that opens on the cytosolic surface of GPCRs on agonist activation. ICL2 interacts with “rim” residues that flank H5 when it associates with GPCRs. The subtle changes observed in ICL4, depending on the nature of the interacting G protein, are powerful drivers of G protein association and specificity. In our molecular dynamics simulation of CLR, a very early event was a subtle movement of the proximal end of ICL1 followed by rearrangements of the TM2/ICL2 interface and then ICL4. We suggest that these underlie the actions of ICL1 in G protein-coupling. Some caution is needed in extrapolating between the molecular dynamics simulation as structural data suggests large scale movements of the cytoplasmic face of CLR require a G protein to stabilise them (14); however, the simulations may have value in reflecting the conformational changes that are required for the G protein interaction.

It is possible to suggest functions for some individual amino acids within ICL1. The majority of crystal structures show that the side chain of L169^12.50^, (residue 169 in both CGRP and GCGR) in the middle of the loop points towards H8 (**Figure 9**). Thus L^12.50^ seems to be required to stabilise the H8 interaction, consistent with our observations that almost any mutation here reduces receptor expression. Residue 12.49 is usually basic and its sidechain points beneath the receptor; where a G protein is present it interacts with D312 of Gβ (**Figures 9B, E**). In the CGRP receptor, 12.49 is serine (perhaps connected to D312 and H8 by water molecules) and, interestingly, its mutation to arginine increases CGRP potency. This may work by promoting G protein interaction. K^12.48^ of CLR also H-bonds to D52 and T50 of Gβ. In all class B1 GPCR structures, the absolutely conserved C^2.44b^ faces towards H8 and R^2.46b^, where its side chain is close to E8.49b, perhaps helping to stabilise the ionic interaction between the arginine and glutamic acid. The polarizability of the sulphur atom in cysteine may be significant here and explain the absolute conservation of the residue. There are also receptor-specific contacts for this residue. For the CGRP and active CRF1 receptors, its sidechain is orientated to Gαs, although still close to R^2.46b^. In the inactive GCCR (5EE7) and GLP-1R (5VEW), it also H-bonds to a water molecule in the cleft between H8 and ICL1. Alanine mutation of C^2.44b^ in the PTH1 receptor results in loss of receptor expression (57), further suggestive of a role in all class B1 GPCRs. In the GCGR where the 2.45b is the shorter threonine a bridging water molecule allows a hydrogen bond between the backbone amides of 2.45b, 2,46b and the phenolic hydroxyl of Y248^3.53b^ (**Figure 9H**). In the CRF1R, L150^2.45b^ mediates hydrophobic interactions.

For all class B1 GPCRs, there are a complex set of interactions between the absolutely conserved R^2.46b^ at the ICL1/TM2 junction and E^8.49b^ and N^7.61b^ at the H8/TM7 junction in the inactive receptors, probably involving water molecules (52) (**Figures 9B, F**). In the active receptors, these contacts undergo rearrangement to include Q390 and E392 at the C-terminus of H5 of Gαs (**Figure 8**). This pattern of interaction is broadly similar in the PTH1, GLP-1, CGRP and calcitonin receptors (6NIY) (8, 53, 54), although the structures show differences in the orientation of the side chains.

Whilst it is possible explain some of the mutagenesis data in this study, it is hard to convincingly rationalise every result. Perhaps the most economical explanation is to suggest that mutations anywhere along ICL1 have the potential to change its conformation and so indirectly alter its interactions with ICL2 and ICL4. For this to be plausible, ICL1 must have a degree of flexibility. The molecular dynamics simulation suggests this is possible. However, the GPCR structures also speak to this issue. Perhaps not surprisingly, in the crystal structures ICL1 has a high B-factor, consistent with mobility. However, for the GCGR, two distinct conformations are seen, differing in their orientation of L169^12.50^ (**Figures 9D, E**). For the CRFR1 structures, in the inactive 4Z9G, H8 cannot be resolved and the sidechain of L^12.50^ also points between TM1 and 2; it is in a single turn of a helix at the proximal end of ICL1. However, in the active structures (6PB0, 6P9X), where H8 is seen, the helix is lost and L^12.50^ now faces H8. Thus, ICL1 is not rigid and mutations could have unpredictable effects on its structure. Hydrogen-deuterium exchange has demonstrated that even in the absence of CGRP, ICL1 of CLR/RAMP1, with the other intracellular loops, has a high rate exchange, consistent with flexibility (14).

It is worth considering how H8 is adapted as a partner to ICL1. E^8.49b^ of H8 faces centrally into ICL1 (**Figures 9A–E**); its presence may partially explain why the introduction of glutamate anywhere in ICL1 is deleterious as this is likely to result in ionic repulsion. The small increase in basal activity seen in the GCGR when it is substituted by alanine may be explained by the presence of an interaction between it and R346 at the base of TM6 seen in the inactive receptor (5EE7), which helps constrain the base of the receptor in a closed form. E^8.49b^ is probably important in maintaining the orientation of H8 as it can interact directly or indirectly to Q/N ^7.61b^ in both active and inactive receptors (**Figures 9A, B, D, F**), hence explaining why alanine mutagenesis reduces receptor expression. Apart from the junction with TM7, the rest of H8 appears to be of little importance for G protein coupling but not for ERK1/2 activation. This presumably reflects the role that H8 plays in β-arrestin recruitment and subsequent ERK1/2 phosphorylation (58–61)

In the calcitonin and CRF1 receptors, splice variants exist in ICL1 (31). In this study, we show that the insertion changes receptor bias of the CRF1 receptor. For the calcitonin receptor there is reduced coupling to both cAMP and Ca^2+^ signaling, although it is not clear if there is any change in bias. It is not possible to provide any detailed mechanistic explanation with excessive speculation; however, it is significant that endogenous variants of ICL1 show differences in bias.

The equivalent of the RKLK motif can be recognized in class A GPCRs, where the ICL1 residues form a loosely conserved [R/H]^1.61^KL[R/H] motif; the H8 motif is E^8.49^-FxxxF (**Figure 1**) (32). ICL1 has been identified as being important for constraining class B1 A and C GPCRs in inactive states (13), perhaps hinting at a wider role the motif may play. ICL1 is in close proximity to the binding sites for a number of allosteric antagonists that bind to the intracellular face of GPCRs (50, 62, 63) and also the probable site of action of pepducins based on ICL1 sequences (64, 65). Given the number of interactions it can make, it may be a useful area for drug targeting.

In conclusion, ICL1 can influence the architecture of the G protein-binding pocket to favour either G_s_ or G_q/11_ coupling, making this an important regulator of G protein specificity. Residues at the distal end of the loop are particularly important and we suggest that they work largely by influencing the interplay between ICL4 and H5 of the G protein Gα subunit.

## Data Availability Statement

The raw data supporting the conclusion of this article will be made available by the authors, on reasonable request.

## Author Contributions

IW, KB, SR, NJR, MH, and JS performed the experiments and modelling. CAR, AJ, DRP, and GL conceived the idea and analysed data. DRP, GL, and CAR wrote the manuscript. All authors provided edits and comments. All authors contributed to the article and approved the submitted version.

## Funding

This work was supported by the following BBSRC grants (BB/M00015X/1 (to GL), BB/M000176/1 (to DRP), and BB/M006883/1 (to C.A.R.), MRC Doctoral Training Partnership MR/J003964/1 (to IW), the BBSRC-funded Midlands Integrative Biosciences Training Partnership (MIBTP) (KB).

## Conflict of Interest

AJ and NJR were employees of Sosei Heptares at the time the work was performed. Neither are now employed by Sosei Heptares.

The remaining authors declare that the research was conducted in the absence of any commercial or financial relationships that could be construed as a potential conflict of interest.

## Publisher’s Note

All claims expressed in this article are solely those of the authors and do not necessarily represent those of their affiliated organizations, or those of the publisher, the editors and the reviewers. Any product that may be evaluated in this article, or claim that may be made by its manufacturer, is not guaranteed or endorsed by the publisher.
