# Supplementary material for: The Role of ICL1 and H8 in Class B1 GPCRs; Implications for Receptor Activation
Source: Front Endocrinol (Lausanne). 2022 Jan 13;12:792912. doi: 10.3389/fendo.2021.792912 (PMC8796428; doi:10.3389/fendo.2021.792912)
Supplement: Supplementary file 1 [file DataSheet_1.pdf]

Table S1. Cell surface expression data for SNAP-tagged CLR ICL1 mutants, co-expressed with RAMP1, as determined via ELISA. Data represented as percentage expression of wild type CLR. n=minimum of 3 triplicate repeats.

	WT Residue							
	**Y165**	**F166**	**K167**	**S168**	**L169**	**S170**	**C171**	**Q172**
**Ala/Leu**	121.6±11.7	110.4± 6.5	55.0±3.7	89.6±8.4	36.2±2.9	101.6±7.2	79.1±7.2	78±2.3

Table S2. Cell surface expression data for SNAP-tagged CLR H8 mutants, co-expressed with RAMP1, as determined via ELISA. Data represented as percentage expression of wild type CLR. Residues changed to Ala except for A393 where Leu was introduced. n=minimum of 3 triplicate repeats.

	WT Residue											
	**N388**	**G389**	**E390**	**V391**	**Q392**	**A393**	**I394**	**L395**	**R396**	**I397**	**L398**	**R399**
**Ala/Leu**	96±4.5	101±6.5	51.2±8.8	93.4±10.5	104.3±12.2	96±3.4	20±5.6	51.34±9.1	82.3±2.3	48±2.4	56.1±7.5	50.15±18

Table S3a: Potency (pEC_{50}) for cAMP production in HEK 293 cells, co-expressing RAMP 1 and alanine CLR ICL1 mutants, and H8 mutants upon CGRP stimulation, normalised with respect to WT CLR.

ICL1

	pEC_{50}[a]	E_{max}[b]	*n*
**WT**	8.90±0.2	102.1±6.4	10
**Y165A**	8.48±0.2	104.7±4.0	6
**F166A**	8.12±0.3	62.9±8.2**	6
**K167A**	8.85±0.2	122.6±6.6	6
**S168A**	8.78±0.2	115.9±7.9	6
**L169A**	8.44±0.2	69.9±6.2*	6
**S170A**	8.85±0.2	129.8±8.3*	6
**C171A**	7.68±0.4**	86.2±10.5	8
**Q172A**	8.35±0.2	68.5±5.7*	8
**R173A**	7.84±0.3**	63.1+6.4**	8

Helix 8

	pEC_{50}[a]	E_{max}[b]	*n*
**WT**	8.34±0.1	99.8±1.4	6
**N388A**	7.23±0.10**	110.7±5.6	5
**G389A**	8.19±0.12	101.5±2.5	6
**E390A**	7.71±0.2*	64.1±2.2*	6
**V391A**	7.52±0.1*	89.8±2.8	10
**Q392A**	8.58±0.1	105.8±2.5	3
**A393L**	8.59±0.1	99.9±2.3	4
**I394A**	8.23±0.2	94.9±4.4	4
**L395A**	7.72±0.1*	100.1±2.9	4
**R396A**	8.36±0.1	105.4±3.3	4
**R397A**	8.05±0.1	44.6±3.8**	4
**N398A**	8.18±0.1	91.4±2.5	4
**W399A**	7.74±0.1*	90.1±3.7	4

Data ± SEM of *n* individual replicates.
[a] Negative logarithm of agonist concentration producing half-maximal response.
[b] Maximal response observed upon CGRP stimulation, as a percentage of that observed for wild type CLR.
Statistical difference between each mutant and wild type CLR was calculated using a one-way ANOVA with Dunnets post-test (*, $p < 0.05$, **, $p < 0.01$, ***, $p < 0.001$, ****, $p < 0.0001$).

Table S3b: Affinity (pKa) and coupling efficacy (log τ) values for cAMP production in HEK 293 cells, co-expressing RAMP 1 and alanine CLR ICL1 mutants, and H8 mutants upon CGRP stimulation, normalised with respect to 100μM forskolin stimulation.

ICL1

	E_{max} [a]	pKa [b]	logτ [c]	*n*
**WT**	38.92±2.4	8.69±0.2	-0.19±0.02	10
**Y165A**	50.78±1.9	8.81±0.2	-0.09±0.03*	6
**F166A**	29.47±1.4*	8.54±0.3	-0.42±0.02***	6
**K167A**	43.90±1.7	8.72±0.2	-0.14±0.02	6
**S168A**	43.37±2.5	8.17±0.2	-0.11±0.04	6
**L169A**	24.39±1.4*	8.13±0.3	-0.50±0.03**	6
**S170A**	55.25±6.4*	8.00±0.2	-0.082±0.03*	6
**C171A**	31.23±2.1	7.57±0.3**	-0.35±0.03***	8
**Q172A**	27.28±1.7*	8.09±0.3	-0.42±0.02***	8

Helix 8

	E_{max} [a]	pKa [b]	Logτ [c]	*n*
**WT**	50.0±0.7	8.39±0.1	-0.17±0.03	6
**N388A**	55.35±2.8	7.13±0.3**	-0.58±0.03**	6
**G389A**	50.5±1.2	8.45±0.2	-0.19±0.01	6
**E390A**	32.2±1.1*	7.72±0.2*	-0.42±0.04*	6
**V391A**	35.0±1.4*	7.76±0.1*	-0.23±0.03	10
**Q392A**	52.4±1.3	8.62±0.1	-0.09±0.04	3
**A393L**	50.0±1.2	8.41±0.1	-0.12±0.02	4
**I394A**	47.45±2.2	8.38±0.1	-0.18±0.03	4
**L395A**	50.0±1.5	7.55±0.1*	-0.12±0.03	4
**R396A**	52.2±1.8	8.24±0.1	-0.08±0.04	4
**R397A**	23.45±4.4*	7.96±0.1	-0.17±0.04	4
**N398A**	46.00±1.3	8.05±0.1	-0.08±0.03	4
**W399A**	45.05±1.8	7.60±0.1*	-0.16±0.03	4

Data ± SEM of *n* individual replicates.

[a] Maximal response observed upon CGRP stimulation, as a percentage of that observed for 100μM forskolin stimulation

[b] Negative logarithm of the equilibrium dissociation constant, as determined using the operational model of agonism (Black and Leff, 1983).

[c] Coupling efficacy parameter as determined using the operational model of agonism (Black and Leff, 1983).

Statistical difference between each mutant and wild type CLR was calculated using a one-way ANOVA with Dunnets post-test (*, p < 0.05, **, p < 0.01, ***, p < 0.001).

Table S4:
Potency (pEC$_{50}$), affinity (pKa) and coupling efficacy (log τ) values for $_{i}Ca^{2+}$ mobilization in HEK 293 cells, co-expressing RAMP 1 and alanine CLR ICL 1 mutants, and H8 mutants upon CGRP stimulation.

ICL1

	**pEC$_{50}$[a]**	**E_{max}[b]**	**pKa[c]**	**logτ[d]**	***n***
**WT**	9.07±0.2	33.1±2.8	8.85±0.2	-0.33±0.04	3
**Y165A**	9.49±0.3	33.1±2.3	9.38±0.1	-0.28±0.03	3
**F166A**	9.03±0.3	33.5±3.7	8.76±0.2	0.32±0.05	3
**K167A**	9.81±0.1*	27.6±2.0	9.61±0.1*	-0.43±0.03	3
**S168A**	8.18±0.3*	21.6±4.0*	8.03±0.2*	-0.60±0.5*	3
**L169A**	6.45±1.0***	40.2±15.0	6.03±0.1***	0.21±0.03**	3
**S170A**	7.22±0.7**	35.1±9.8	6.95±0.3***	-0.31±0.1	3
**C171A**	6.11±1.2***	59.4±17.5**	6.18±0.2***	0.18±0.04**	3
**Q172A**	8.07±0.5**	12.3±2.0**	8.10±0.2**	-0.85±0.04*	3

Helix 8

	**pEC$_{50}$[a]**	**E_{max}[b]**	**pKa[c]**	**Logτ[d]**	***n***
**WT**	9.07±0.02	49.0±2.6	8.84±0.2	-0.31±0.04	7
**E390A**	7.52±0.2***	59.5±3.8*	7.34±0.2***	-0.12±0.08	8
**V391A**	8.81±0.2**	51.0±2.9	8.66±0.2	-0.28±0.04	8
**Q392A**	9.11±0.2	50.0±2.8	8.91±0.1	-0.27±0.04	8
**A393L**	9.40±0.2	49.7±2.03	9.20±0.1	-0.30±0.03	8
**I394A**	N.R.	N.R.	N.R.	N.R.	4
**L395A**	8.90±0.4	43.6±4.6	8.73±0.3	-0.42±0.08	8
**R396A**	8.40±0.2**	56.8±2.8	8.21±0.1*	-0.19±0.04*	8
**R397A**	9.22±0.2	44.7±3.0	9.06±0.2	-0.35±0.05	8
**N398A**	8.52±0.2*	45.0±2.2	8.40±0.2*	-0.33±0.04	8
**W399A**	8.91±0.1	45.5±1.5	8.75±0.1	-0.36±0.02	8

Data ± SEM of *n* individual replicates.
[a] Negative logarithm of agonist concentration producing half-maximal response.
[b] Maximal response observed upon stimulation with 100 μM ionomycin
[c] Negative logarithm of the equilibrium dissociation constant, as determined using the operational model of agonism (Black and Leff, 1983).
[d] Coupling efficacy parameter as determined using the operational model of agonism (Black and Leff, 1983).
Statistical difference between each mutant and wild type CLR was calculated using a one-way ANOVA with Dunnets post-test (*, $p < 0.05$, **, $p < 0.01$, ***, $p < 0.001$).
N.R. denotes no detectable response.

Table S5: Potency (pEC_{50}), affinity (pKa) and coupling efficacy (log τ) values for ERK1/2 activation in HEK 293 cells, co-expressing RAMP 1 and alanine CLR ICL 1 mutants (A), and H8 mutants (B) upon CGRP stimulation.

ICL1

	pEC_{50}[a]	E_{max}[b]	pKa[c]	logτ[d]	*n*
**WT**	7.35±0.3	26.7±5.1	6.49±0.4	-0.0037±0.1	3
**Y165A**	7.67±0.4	13.6±3.1*	7.68±0.5*	-0.70±0.1**	3
**F166A**	7.82±0.3	19.6±3.1*	7.68±0.3*	-0.56±0.08**	3
**K167A**	8.10±0.3**	17.7±2.8*	8.00±0.3**	-0.59±0.08**	3
**S168A**	6.82±0.4*	30.6±7.5	6.78±0.4	-0.35±0.2*	3
**L169A**	6.71±0.3	29.6±6.5	6.51±0.3	-0.32±0.1*	3
**S170A**	6.64±0.6*	34.8±13.9	6.44±0.6	-0.24±0.3*	3
**C171A**	7.00±0.5	32.5±9.3	6.78±0.6	-0.35±0.2*	3
**Q172A**	6.84±0.3*	33.8±5.7	6.69±0.3	-0.27±0.1*	3

Helix 8

	pEC_{50}[a]	E_{max}[b]	pKa[c]	Logτ[d]	*n*
**WT**	7.26±0.1	45.5±1.9	6.96±0.1	0.0042±0.04	5
**E390A**	8.92±0.2***	40.1±1.9	8.67±0.2***	-0.10±0.05*	5
**V391A**	8.41±0.3**	34.7±2.5*	8.20±0.3**	-0.19±0.05*	5
**Q392A**	8.17±0.1***	30.8±1.09**	7.99±0.1***	-0.28±0.03***	5
**A393L**	7.22±0.1	40.5±2.3	6.96±0.2	-0.089±0.05*	5
**I394A**	N.R.	N.R.	N.R.	N.R.	4
**L395A**	7.43±0.3	26.5±2.4**	7.29±0.3	-0.40±0.06***	5
**R396A**	5.30±0.6***	28.6±14.3**	5.14±0.7*	-0.33±0.30*	5
**R397A**	5.46±0.4***	27.6±7.2**	5.29±0.4**	-0.34±0.20*	5
**N398A**	7.38±0.2	16.3±1.2***	7.30±0.2	-0.70±0.05***	5
**W399A**	6.12±0.2***	22.0±2.0*	6.00±0.2**	-0.48±0.05***	5

Data ± SEM of *n* individual replicates.
[a] Negative logarithm of agonist concentration producing half-maximal response.
[b] Maximal response observed upon stimulation with 100 μM PMA
[c] Negative logarithm of the equilibrium dissociation constant, as determined using the operational model of agonism (Black and Leff, 1983).
[d] Coupling efficacy parameter as determined using the operational model of agonism (Black and Leff, 1983).
Statistical difference between each mutant and wild type CLR was calculated using a one-way ANOVA with Dunnets post-test (*, $p < 0.05$, **, $p < 0.01$, ***, $p < 0.001$).
N.R. denotes no detectable response.

Table S6: Potency (pEC_{50}), affinity (pKa) and coupling efficacy (log τ) values for cAMP, $_iCa^{2+}$ and ERK1/2 activation in HEK 293 cells, expressing alanine GCGR ICL1 mutants upon GCG stimulation.

cAMP

	pEC_{50}[a]	E_{max}[b]	pKa[c]	logτ[d]	*n*
**WT**	9.94±0.2	44.2±2.2	9.83±0.1	-0.14±0.003	3
**G165A**	9.82±0.3	39.4±2.8	9.61±0.1	-0.22±0.004	3
**L166A**	10.3±0.2	40.8±1.9	10.1±0.1	-0.2±0.004	3
**S167A**	9.77±0.2	41.9±0.16	9.73±0.2	-0.19±0.004	3
**K168A**	9.92±0.2	45.1±2.6	9.69±0.2	-0.12±0.005	3
**L169A**	9.71±0.3	24.6±2.0**	9.55±0.3	-0.54±0.005	3
**H170A**	9.83±0.2	45.3±2.3	9.71±0.3	-0.12±0.005	3
**C171A**	N.R.	N.R.	N.R.	N.R.	3
**T172A**	9.93±0.3	29.9±2.3*	10.2±0.2	-0.44±0.004	3

$_iCa^{2+}$

	pEC_{50}[a]	E_{max}[e]	pKa[c]	logτ[d]	*n*
**WT**	6.5±0.24	48.8±4.9	6.21±0.1	-0.056±0.05	3
**G165A**	6.66±0.2	41.2±2.8	6.51±0.2	-0.197±0.06	3
**L166A**	6.3±0.2	41.6±2.9	6.20±0.2	-0.188±0.09	3
**S167A**	6.54±0.3	33.3±3.6*	6.42±0.3	-0.352±0.09**	3
**K168A**	6.49±0.4	22.7±3.0**	6.40±1.3	-0.607±0.24***	3
**L169A**	6.43±0.2	17.8±0.9**	6.49±0.7	-0.767±0.20***	3
**H170A**	6.21±0.3	21.7±2.4**	6.19±1.0	-0.638±0.22***	3
**C171A**	N.R.	N.R.	N.R.	N.R.	3
**T172A**	6.34±0.4	25.3±3.6**	6.17±0.6	-0.537±0.15***	3

ERK1/2

	pEC_{50}[a]	E_{max}[f]	pKa[c]	logτ[d]	*n*
**WT**	8.34±0.3	92.8±11.1	7.77±0.5	-0.231±0.098	3
**G165A**	8.56±0.2	92.9±7.2	8.07±0.9	-0.231±0.087	3
**L166A**	7.22±0.4**	101.1±21	6.16±1.0**	-0.112±0.139	3
**S167A**	6.79±0.4**	119.0±28*	5.66±0.9**	-0.062±0.148**	3
**K168A**	8.12±0.3	86.4±7.7	7.94±0.5	-0.299±0.090	3
**L169A**	7.19±0.4**	74.9±18	6.26±0.8	-0.295±0.198	3
**H170A**	7.20±0.3**	101.1±10	7.35±1.0	-0.255±0.100	3
**C171A**	N.R.	N.R.	N.R.	N.R.	3
**T172A**	8.25±0.5	50.4±10.7**	7.94±0.6	-0.615±0.133**	3

Data ± SEM of *n* individual replicates.
[a] Negative logarithm of agonist concentration producing half-maximal response.
[b] Maximal response observed upon stimulation with 100 µM Forskolin.
[c] Negative logarithm of the equilibrium dissociation constant, as determined using the operational model of agonism (Black and Leff, 1983).
[d] Coupling efficacy parameter as determined using the operational model of agonism (Black and Leff, 1983).
[e] Maximal response observed upon stimulation with 100 µM ionomycin.
[f] Maximal response observed upon stimulation with 100 µM PMA.
Statistical difference between each mutant and wild type GCGR was calculated using a one-way ANOVA with Dunnets post-test (*, $p < 0.05$, **, $p < 0.01$, ***, $p < 0.001$).
N.R. denotes no detectable response.

Table S7: Potency (pEC_{50}), affinity (pKa) and coupling efficacy (log τ) values for cAMP, $_iCa^{2+}$ and ERK1/2 activation in HEK 293 cells, expressing CRFR1a and CRFR1b upon stimulation with CRF.

	pEC_{50}[a]	E_{max}[b]	pKa[c]	logτ[d]	*n*
		**cAMP**			
**CRFR1a**	7.66±0.2	37.38±3.46	7.39±0.21	-0.22±0.06	3
**CRFR1b**	7.76±0.24	28.68±2.78	7.83±0.24	-0.41±0.06	3
		**$_iCa^{2+}$**			
**CRFR1a**	10.03±0.23	53.82±3.7	9.70±0.23	0.06±0.08	3
**CRFR1b**	6.71±0.42***	32.17±7.1*	6.57±0.47***	-0.42±0.16***	3
		**ERK1/2**			
**CRFR1a**	8.62±0.28	29.63±1.89	8.53±0.28	-0.67±0.06	3
**CRFR1b**	8.06±0.25	24.81±2.32	8.00±0.26	-0.62±0.07	3

Data ± SEM of *n* individual replicates.
[a] Negative logarithm of agonist concentration producing half-maximal response.
[b] Maximal response observed upon stimulation with 100 μM Forskolin (cAMP), 100 μM ionomycin (iCa^{2+}) or 100 μM PMA (ERK1/2).
[c] Negative logarithm of the equilibrium dissociation constant, as determined using the operational model of agonism (Black and Leff, 1983).
[d] Coupling efficacy parameter as determined using the operational model of agonism (Black and Leff, 1983).
Statistical difference between CRFR1a and CRFR1b was calculated using a one-way ANOVA with Dunnets post-test (*, $p < 0.05$, **, $p < 0.01$, ***, $p < 0.001$).

Figure S1: Multiple sequence alignment of ICL1 (left) and H8 (right) of human Class B1 GPCRs

CALRL_HUMAN	YFK$^{12.48}$SLSCQR	NGE$^{8.49b}$VQAILRRNW
CALCR_HUMAN	FFR$^{12.48}$KLGCQR	NEV$^{8.49b}$QTTVKRQYW
CRFR1_HUMAN	RLR$^{12.48}$SIRCLR	NSE$^{8.49b}$VRSAIRKRW
GCGR_ HUMAN	GLS$^{12.48}$KLHCTR	NKE$^{8.49b}$VQSELRRRW
GLP1R_HUMAN	GFR$^{12.48}$HLHCTR	NEV$^{8.49b}$QLEFRKSRW
GLP2R_HUMAN	FLR$^{12.48}$KLHCTR	NGE$^{8.49b}$VKAELRKYW
GIPR_ HUMAN	LFR$^{12.48}$RLHCTR	NKE$^{8.49b}$VQSEIRRGW
VIPR1_HUMAN	LFR$^{12.48}$KLHCTR	NGE$^{8.49b}$VQAELRRKW
PACR_ HUMAN	RFR$^{12.48}$KLHCTR	NGE$^{8.49b}$VQAEIKRKW
VIPR2_HUMAN	LFR$^{12.48}$KLHCTR	NSE$^{8.49b}$VQCELKRKW
SCTR_ HUMAN	AFR$^{12.48}$RLHCTR	NGE$^{8.49b}$VQLEVQKKW
PTH2R_HUMAN	YFR$^{12.48}$RLHCTR	NGE$^{8.49b}$VQAEVKKMW
GHRHR_HUMAN	ALR$^{12.48}$RLHCPR	NQE$^{8.49b}$VRTEISRKW
PTH1R_HUMAN	YFR$^{12.48}$RLHCTR	NGE$^{8.49b}$VQAEIKKSW

Multiple sequence alignment (left) of ICL1 and flanking residues and H8 and its junction with helix 7 for human family B GPCRs. The start of ICL1 at 12.48 and H8 at 8.49b are marked.

Supplementary Video 1. Molecular dynamics simulation of the CLR during inactive to active transition: a) view of the cytoplasmic surface, ICL1 is in orange at the bottom right; b) view of the TM bundle. The side chains are S12.49, L12.50, C2.44, R2.46, N3887.61b and E3908.49, as in Fig 8A.

Supplementary Video 2. Molecular dynamics simulation of the GCGR during inactive to active transition: a) view of the cytoplasmic surface; ICL1 is in orange at the bottom right; b) view of the TM bundle.
